# Cardiac Injury and Clinical Course of Patients With Coronavirus Disease 2019

**DOI:** 10.3389/fcvm.2020.00147

**Published:** 2020-08-25

**Authors:** Yushi Wang, Yang Zheng, Qian Tong, Lihui Wang, Guorui Lv, Ziwei Xi, Wei Liu

**Affiliations:** ^1^Departments of Cardiology, First Teaching Hospital of Jilin University, Changchun, China; ^2^Hepatic-Gastroenterology, First Teaching Hospital of Jilin University, Changchun, China; ^3^Department of Cardiology, BeiJing Anzhen Hospital, Capital Medical University, Changchun, China

**Keywords:** COVID-19, cardiac injury, troponin, mortality, disease course

## Abstract

**Background:** Cardiac injury is recognized as one of the most common critical complications during exacerbation of coronavirus disease 2019 (COVID-19). This study aimed to investigate the effect of cardiac injury on the clinical course of COVID-19 and to examine its potential mechanism and treatments.

**Methods and Results:** A total of 222 hospitalized patients with COVID-19 from Wuhan were selected for the study during February 10 to March 28, 2020. Demographic, laboratory, and clinical data on admission and during hospitalization were compared between patients with COVID-19 with or without cardiac injury. On admission, cardiac injury (*n* = 29) was associated with advanced age, more underlying coronary artery disease, and a lower Pao_2_. Troponin levels were correlated with inflammatory markers (C-reactive protein: *r* = 0.348, *P* < 0.001; interleukin 6: *r* = 0.558, *P* < 0.001) and d-dimer levels (*r* = 0.598, *P* < 0.001). During hospitalization, another six patients suffered from cardiac injury and cardiac injury (*n* = 35), resulting in higher rates of ventilation (invasive: 51.4 vs. 1.6%, *P* < 0.001; non-invasive: 31.4 vs. 1.1%, *P* < 0.001) and mortality (54.3 vs. 1.1%, *P* < 0.001). Cardiac injury on admission was a predictive factor for mortality (adjusted hazard ratio = 4.73, 95% confidence interval = 1.35–16.63, *P* = 0.015). Receiver operating characteristic curve analysis showed that, on admission, a troponin level of 36.35 pg/mL was predictive for mortality with a sensitivity of 73.7% and a specificity of 92.1%.

**Conclusions:** Cardiac injury complicates the disease course and increases the mortality rate of COVID-19. Troponin levels should be checked at admission and during hospitalization for triage, better monitoring, and managing those with COVID-19, especially in the most severe patients.

**Condensed Abstract:** Cardiac injury is not uncommon in COVID-19. In a cohort of 222 patients with COVID-19, cardiac injury was found in 29 patients on admission and in another 6 patients during hospitalization. The admission level of troponin was well-correlated with inflammatory factors and d-dimer levels and strongly predicted mortality. Cardiac injury is a manifestation secondary to hypoxia and systemic infection, but which nevertheless further complicates the disease course and increases the mortality rate. Troponin levels should be checked at admission and during hospitalization for triage, better monitoring, and managing those with COVID-19, especially in the most severe patients.

The escalating coronavirus disease 2019 (COVID-19) pandemic has evolved into a major public health crisis. There is a wide range of variation in reported mortality rates of COVID-19. The mortality rate of COVID-19 is 2.3% (1) according to a report from the Chinese Center for Disease Control and Prevention, but it is close to 10% in European countries, such as Italy and Spain (2). The mortality rate is <1% in population-based studies that have included mild and asymptomatic cases. In addition to severe respiratory distress, an overwhelming systemic inflammatory reaction that leads to multiple organ system failure appears to be the underlying cause for mortality of this disease (3). Cardiac involvement is one of the most common critical complications during exacerbation of COVID-19, especially in patients with underlying chronic cardiovascular disease (4, 5). Reports from China have shown that elevated troponin levels are associated with a worse outcome, and other biomarkers include lymphocytopenia and elevation of alanine aminotransferase, d-dimer, or interleukin 6 (IL-6) levels (6, 7). Patients with COVID-19 with underlying coronary artery disease and new cardiac injury showed the highest mortality rate (4). A recent case series of 18 patients with COVID-19 and ST-segment elevation cardiac injury from New York showed a mortality rate of up to 72% (8). Patients with non-coronary cardiac injury have a relatively higher death rate than those who are diagnosed with acute myocardial infarction. Early recognition of cardiac injury, close monitoring, and managing heart dysfunction may prevent excessive morbidity and mortality and improve prognosis of patients with COVID-19.

In a Chinese cohort of patients in Wuhan with COVID-19 who have not previously been studied, we investigated the clinical characteristics of these patients and the effect of COVID-19 on the clinical course and mortality rate from cardiac injury. We also discuss the potential mechanism and treatment strategy with the aim of decreasing the rate of fatality in patients with a severe form of COVID-19 infection.

## Methods

### Study Population

All of the studied patients were hospitalized in the Sino-French New City Branch of Tongji Hospital in Wuhan, China, which was the epicenter in the initial COVID-19 outbreak. This branch was set up to accept the most critically ill patients since February 10, 2020. Doctors were urgently recruited from several provinces in China. The patients who were selected for the study were cared for by the team that originated from Jilin Province, China. Adult patients who were identified as having laboratory-confirmed COVID-19 infection were enrolled. We excluded patients whose cardiac enzymes were not checked and those with insufficient laboratory data.

All patients were admitted for severe pneumonia due to COVID-19 with unstable vital signs (saturation <93% at room air), or they had severe chronic underlying diseases. COVID-19 pneumonia was confirmed in all of the patients by reverse transcriptase–polymerase chain reaction (RT-PCR) assay or serological testing. All patients presented with positive computed tomographic findings (ground-glass opacities and consolidation with or without vascular enlargement, interlobular septal thickening, and air bronchogram sign) (9).

### Laboratory Testing

Nasal pharyngeal swabs or upper or lower respiratory tract samples were collected in all of the patients to test for severe acute respiratory syndrome coronavirus 2 (SARS-CoV-2) by nucleic acid RT-PCR. On admission, all patients had a comprehensive laboratory examination, including measurement of B-type natriuretic peptide (BNP) (normal limit = <241 pg/mL), high-sensitivity troponin I (hs-TnI) (normal limit = <34.2 pg/mL), blood cytology, biochemistry, blood gases, a coagulation panel, and inflammatory indicators. The entire laboratory test was repeated at least once a week or more frequently if the patient's condition was unstable.

### Definitions

Cardiac injury was defined as an increase in troponin levels above the 99th percentile upper reference limit, regardless of new abnormalities in an electrocardiogram (ECG) and echocardiography (10). The diagnosis of myocardial infarction, especially type 2 myocardial infarction, was not made because of insufficient data in the period of initial urgency when medical teams for COVID-19 were being assembled. Acute respiratory distress syndrome (ARDS) was defined according to the Berlin definition. Acute kidney injury was identified according to the Kidney Disease: Improving Global Outcomes definition.

### Treatment

Supportive treatment and antiviral medication (Chinese herbal medication and antiviral medication, including lopinavir/ritonavir, chloroquine phosphate, and arbidol) were provided to patients at the discretion of the individual physician. Low-dose (40 mg) methylprednisolone was administered for 3 to 5 days for unstable patients with persistent symptoms (febrile, increasing dyspnea, desaturation).

High-flow nasal catheter oxygenation or non-invasive mechanical ventilation was initiated when the patient's respiratory distress and/or hypoxemia was not improved after standard oxygen therapy. If the patient's condition did not respond to these non-invasive measures or even deteriorated within a short period of time (1–2 h), tracheal intubation and invasive mechanical ventilation were used.

Ventilation in the prone position was performed for more than 12 h per day. Indications of extracorporeal membrane oxygenation (ECMO) included the following: (1) When Fio_2_ was >90% and the oxygenation index was <80 mmHg for longer than 3 to 4 h; and (2) for patients with only respiratory failure when the airway platform pressure was ≥35 cmH_2_O, the VV-ECMO mode was preferred, and if circulatory support was required, the VA-ECMO mode was used.

### Endpoints

The primary endpoint was death or recovery. Follow-up was until March 28, 2020. Patients were considered as recovered and ready for discharge if the following criteria were met: temperature returned to normal for more than 3 days; respiratory symptoms were resolved; pulmonary imaging showed considerable resolution of inflammation; and there were two consecutive negative nuclei acid tests on respiratory tract samples, such as sputum and nasopharyngeal swabs (sampling interval of at least 24 h apart).

The data were retrospectively collected by two examiners from electronic medical record and were mutually checked for accuracy. All of the data were analyzed by investigators who were blinded to this study.

The protocol of this study was approved by the ethics committee of the First Teaching Hospital of Jilin University. Written informed consent was waived by the Ethics Commission for Emerging Infectious Diseases.

### Statistical Analysis

Continuous variables are expressed as median [interquartile range (IQR)]. Categorical variables are presented as numbers and percentages. Continuous values were compared by the Mann–Whitney *U*-test. Comparison of categorical variables was performed by the χ^2^ test or Fisher exact test as appropriate. The Spearman correlation coefficient was used to assess the linear correlation between hs-TnI levels and other laboratory results.

Receiver operating characteristic (ROC) curve analysis was used for predicting mortality and identifying the optimal cutoff value of plasma hs-TnI levels on the basis of Youden *J* statistic. Optimal cutoff values were defined as the points on the ROC curve where Youden index (sensitivity + specificity – 1) was the highest. Survival curves were plotted using the Kaplan–Meier method and compared between patients with cardiac injury and those without cardiac injury on admission using the log-rank test. The hazard ratio (HR) and 95% confidence interval (CI) were calculated using multivariate Cox regression models to identify independent predictors of all-cause mortality during hospitalization. To avoid overfitting in the model, five variables that have been reported to be associated with clinical outcomes by previous studies were chosen for multivariable analysis, including plasma hs-TnI levels, the percentage of lymphocytes, d-dimer levels, and IL-6 levels on admission, and age.

All reported *P*-values were two-sided, and *P* < 0.05 was considered statistically significant for all analyses. Statistics were calculated using IBM SPSS Statistics version 25 (IBM Corp., Armonk, NY, USA). Statistical charts were generated using Excel 2016 (Microsoft, Redmond, WA) or Prism 8 (GraphPad Software Inc., San Diego, CA, USA).

## Results

### Baseline Characteristics

A total of 222 patients with sufficient medical information were included in the final analysis. The median age of the patients was 63.0 years (IQR = 50.0–69.0 years), and 113 (50.9%) were men.

### Cardiac Injury in Patients With COVID-19 Infection on Admission

Patients with elevated hs-TnI levels (*n* = 29) were older [median (IQR) = 70.0 (65.5–80.0] vs. 60.5 (48.0–67.0) years; *P* < 0.001], more likely to have hypertension (37.9 vs. 19.8%; *P* = 0.029) and coronary artery disease (24.1 vs. 3.7%; *P* = 0.001), and had a lower Pao_2_ (92.58 ± 2.42 vs. 88.82 ± 9.98 mmHg; *P* < 0.001) compared with patients with normal hs-TnI levels (*n* = 187) (Table 1). The baseline characteristics and laboratory results are shown in Table 1. The white blood cell count [median (IQR) = 6.35 (5.38–10.21) vs. 5.7 (4.2–6.9) × 10^9^/L; *P* < 0.001], neutrophil percentage [median (IQR) = 85.7% (74.3–92.3%) vs. 66.2% (56.3–75.0%); *P* < 0.001], and erythrocyte sedimentation rate [median (IQR) = 56.5 (36.0–87.0) vs. 30 (14–58) mm/h; *P* < 0.001] were significantly higher in patients with elevated hs-TnI levels than in those with normal hs-TnI levels. However, the lymphocyte percentage was significantly lower in patients with elevated hs-TnI levels than in those with normal hs-TnI levels [median (IQR) = 8.9% (5.2–13.5%) vs. 23.05% (15.8–31.5%), *P* < 0.001]. Patients with elevated hs-TnI levels had a lower estimated glomerular filtration rate [median (IQR) = 71.6 (44.1–96.35) vs. 94.5 (77.8–105.4) mL/min · 1.73 m^2^, *P* = 0.003] and albumin levels [median (IQR) = 28.7 (23.8–31.3) vs. 36.3 (32.2–40.3) g/dL, *P* < 0.001] and higher aspartate aminotransferase levels [median (IQR) = 28.5 (23.25–46.25) vs. 23 (18–32) U/L, *P* = 0.033] and CK-MB levels [median (IQR) = 132 (55–294) vs. 61 (36.5–95.0) μg/L, *P* = 0.049] compared with patients with normal hs-TnI levels. High-sensitivity C-reactive protein levels [median (IQR) = 78.9 (10.2–11.4) vs. 5.3 (1.5–31.5) mg/dL, *P* = 0.009] as an inflammatory biomarker were significantly higher in patients with elevated hs-TnI levels than in those with normal hs-TnI levels. Moreover, patients with elevated hs-TnI levels had significantly higher levels of BNP [median (IQR) = 1,468 (382.5–5,651.5) vs. 65.0 (36.5–185.0) pg/mL, *P* < 0.001] and d-dimer [median (IQR) = 4.99 (2.3–21.0) vs. 0.6 (0.3–1.3) μg/mL, *P* < 0.001] than those with normal hs-TnI levels.

**Table 1:** Baseline characteristics and laboratory results of patients with COVID-19.

**Characteristic**	**All (*n* = 222)**	**No Cardiac injury (*n* = 187)**	**Cardiac injury (*n* = 29)**	***P*-value comparing 29 to 187**	**New cardiac injury during hospitalization (*n* = 6)**	***P*-value comparing 6 to 187**
Age, median (IQR), years	63 (50, 69)	60.5 (48.0–67.0)	70 (65.5, 80.0)	<0.001	65.5 (65.0–70.5)	0.074
Gender (male), *n* (%)	113 (50.9)	96 (53.6)	12 (41.4)	0.221	5 (83.3)	0.223
Hypertension, *n* (%)	51 (23.0)	37 (19.8)	11 (37.9)	0.029	3 (50.0)	0.105
Diabetes, *n* (%)	30 (13.5)	23 (12.3)	7 (24.1)	0.086	0 (0.0)	1.000
Coronary artery disease, *n* (%)	14 (6.7)	7 (3.7)	7 (24.1)	0.001	0 (0.0)	1.000
Thyroid, *n* (%)	9 (4.1)	7 (3.7)	2 (6.9)	0.429	0 (0.0)	1.000
ACEI/ARB, *n* (%)	8 (3.6)	6 (3.2)	2 (6.9)	0.328	0 (0.0)	1.000
CCB, *n* (%)	13 (5.9)	9 (4.8)	3 (10.3)	0.226	1 (16.7)	0.276
**Laboratory results on admission, median (IQR)**						
White blood cell × 10^∧^9/L	5.82 (4.34, 7.17)	5.7 (4.2–6.9)	6.35 (5.38, 10.21)	<0.001	5.1 (4.7–7.8)	0.955
Neutrophil, %	68.0 (57.7, 80.0)	66.2 (56.3–75.0)	85.7 (74.3, 92.3)	<0.001	82.0 (81.0–85.2)	0.003
Lymphocyte, %	21.3 (12.25, 29.35)	23.4 (15.8–31.5)	8.9 (5.2, 13.5)	<0.001	11.6 (10.0–12.5)	0.004
Erythrocyte sedimentation rate, mm/h	34 (16, 63)	30.0 (14.0–58.0)	56.5 (36.0, 87.0)	<0.001	50.0 (37.8–69.0)	0.137
Urea, mg/dL	4.9 (3.85, 6.6)	4.5 (3.8–6.2)	7.4 (4.5, 11.78)	0.023	7.8 (5.5–10.1)	0.044
Creatinine, μmoI/L	71 (60, 88)	70.0 (60.0–87.0)	83 (61, 109)	0.076	66.5 (51.8–91.0)	0.751
eGFR, mL/(min·1.73 m2)	92.85 (74.92, 104)	94.5 (77.8–105.4)	71.6 (44.1, 96.35)	0.003	79.4 (67.2–91.5)	0.142
ALT, U/L	25 (16, 42)	26.0 (16.8–43.0)	25.5 (14.75, 37)	0.733	21.0 (18.0–24.0)	0.399
AST, U/L	24 (18.5, 32.5)	22.5 (18.0–32.0)	28.5 (23.25, 46.25)	0.033	24.0 (21.0–65.0)	0.298
TBIL, μmol/L	9.8 (7.1, 12.02)	9.5 (7.0–11.4)	10.60 (8.03, 13.88)	0.143	18.3 (13.3–22.6)	0.002
DBIL, μmol/L	3.9 (2.93, 5.2)	3.6 (2.9–4.7)	4.9 (3.43, 5.98)	0.031	10.4 (6.2–13.6)	0.0001
IBIL, μmol/L	5.3 (4.2, 7.1)	5.3 (4.2–6.8)	5.4 (4.28, 7.8)	0.813	8.1 (7.1–9.7)	0.042
LDH, U/L	237.5 (191.25, 314.5)	227.0 (189.0–290.5)	342 (205.5, 480)	0.012	382.0 (265.0–613.0)	0.034
Total cholesterol, mg/dL	4.08 (3.38, 4.82)	4.2 (3.6–4.9)	3.67 (2.83, 4.47)	0.044	3.6 (3.4–3.6)	0.115
Triglyceride, mg/dL	1.35 (1.02, 2.0)	1.3 (1.0–2.0)	1.50 (0.98, 2.33)	0.909	1.5 (1.4–1.6)	0.811
HDL, mg/dL	1.0 (0.81, 1.18)	1.0 (0.8–1.2)	0.92 (0.74, 1.05)	0.082	0.8 (0.7–0.9)	0.157
LDL, mg/Dl	2.63 (1.96, 3.03)	2.7 (2.0–3.1)	1.85 (1.41, 2.84)	0.024	1.9 (1.6–2.0)	0.029
Creatine kinase–MB fraction, μg/L	65.5 (35.75, 129.0)	61.0 (36.5–95.0)	132 (55, 294)	0.049	84.0 (31.5–154.5)	0.932
Potassium, mEq/L	4.29 (3.98, 4.61)	4.3 (4.0–4.6)	4.49 (3.77, 5.09)	0.297	4.4 (4.0–4.7)	0.760
Sodium, mEq/L	140.2 (138.3, 142.75)	139.9 (138.5–141.9)	141.15 (137.88, 144.9)	0.098	144.1 (141.3–144.1)	0.143
Ferritin, μg/L	565 (287.3, 1,109.5)	545.5 (269.2–936.0)	862 (453.8, 1,264.5)	0.123	2,305.0 (2,128.0–8,288.5)	0.006
HCO_3_, mEq/L	24.55 (22.25, 26.08) ± 3.24	24.7 (23.0–26.1)	24.0 (20.15, 24.85)	0.051	26.2 (24.0–27.9)	0.393
C-reactive protein, mg/dL	10.2 (1.6, 49.8)	5.3 (1.5–31.5)	78.9 (10.2, 11.4)	0.009	124.8 (52.2–218.3)	0.005
BNP, pg/mL	115.5 (52.25, 668.0)	65.0 (36.5–185.0)	1,468 (382.5, 5,651.5)	<0.001	336.0 (236.0–734.5)	0.007
Albumin, g/dL	35.45 (31.07, 39.80)	36.3 (32.2–40.3)	28.7 (23.8, 31.3)	<0.001	33.4 (30.9–34.2)	0.134
d-Dimer, μg/mL	0.97 (0.41, 2.28)	0.6 (0.3–1.3)	4.99 (2.31, 21.0)	<0.001	12.2 (3.1–21.0)	0.002
IL-6, pg/mL	9.66 (2.94, 36.35)	7.4 (2.6–19.5)	43.39 (10.9, 108.01)	<0.001	72.8 (58.6–293.7)	<0.001
Pao_2_, mean ± SD, mmHg	90.56 ± 6.85	92.58 ± 2.42	88.82 ± 9.98	<0.001	85.06 ± 13.88	<0.001

*ACEI, angiotensin-converting enzyme inhibitor; ARB, angiotensin receptor blocker; CCB, calcium channel blocker; eGFR, estimated glomerular filtration rate; ALT, alanine transaminase; AST, aspartate aminotransferase; TBIL, total bilirubin; DBIL, direct bilirubin; IBIL, indirect bilirubin; LDH, lactic dehydrogenase; HDL, high density lipoprotein; LDL, low-density lipoprotein; BNP, B-type natriuretic peptide; IL-6, interleukin 6*.

### Correlations of the Baseline Troponin Level With Baseline Levels of Laboratory Biomarkers

**Figure 1:**
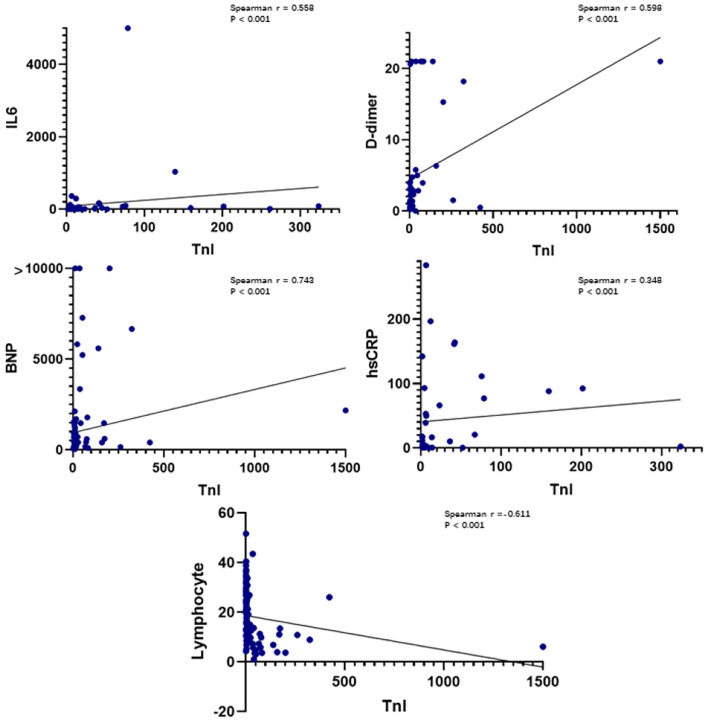
Correlations between plasma hs-TnI levels on admission and IL-6 levels, d-dimer levels, lymphocytes, hs-CRP levels, and BNP levels. IL-6, interleukin 6; BNP, B-type natriuretic peptide; CRP, C-reactive protein; hs-TnI, high-sensitivity troponin I.

Plasma hs-TnI levels were positively correlated with plasma IL-6 levels (Spearman *r* = 0.558, *P* < 0.001), plasma high-sensitivity C-reactive protein levels (Spearman *r* = 0.348, *P* < 0.001), plasma d-dimer levels (Spearman *r* = 0.598, *P* < 0.001), and plasma BNP levels (Spearman *r* = 0.743, *P* < 0.001) (Figure 1). Plasma hs-TnI levels were negatively correlated with the lymphocyte percentage (Spearman *r* = −0.611, *P* < 0.001).

### Patients With Cardiac Injury During Hospitalization

Comparison of complications, treatment, and outcomes between patients with and those without cardiac injury during hospitalization is shown in Table 2. During hospitalization, six patients had newly developed elevation in troponin levels, and a total of 35 patients were diagnosed with cardiac injury. Patients with cardiac injury were significantly more likely to develop complications, including acute kidney injury (17.1 vs. 1.1%, *P* < 0.001) and ARDS (60.0 vs. 2.1%, *P* < 0.001) compared with those without cardiac injury. Results of the comparison of baseline characteristics between patients with newly developed cardiac injury during hospitalization (*n* = 6) and patients without cardiac injury (*n* = 187) are presented in Table 1, which suggested patients with newly developed cardiac injury had higher baseline levels of hs-C-reactive protein, d-dimer, IL-6, ferritin and neutrophil percentage, and lower level of lymphocyte percentage.

**Table 2:** Complications, treatment, and clinical outcome in patients with COVID-19 during hospitalization.

**Characteristic**	**All (*n* = 222)**	**No cardiac injury (*n* = 187)**	**Cardiac injury (*n* = 35)**	***P-*value**
Complication				
AKI, *n* (%)	8 (3.6)	2 (1.1)	6 (17.1)	<0.001
ALF, *n* (%)	12 (5.4)	8 (4.3)	4 (11.4)	0.086
ARDS, *n* (%)	25 (11.3)	4 (2.1)	21 (60.0)	<0.001
Therapy				
Glucocorticoid, *n* (%)	62 (27.9)	44 (23.5)	18 (51.4)	0.001
Inotropics, *n* (%)	10 (4.5)	1 (0.5)	9 (25.7)	<0.001
LMWH, *n* (%)	15 (6.8)	4 (2.1)	11 (31.4)	<0.001
IVGc, *n* (%)	28 (12.6)	13 (7.0)	15 (42.9)	<0.001
Antivirus, *n* (%)	20 (9.0)	13 (7.0)	7 (20.0)	<0.001
Non-invasive mechanical ventilation, *n* (%)	13 (5.9)	2 (1.1)	11 (31.4)	<0.001
Invasive mechanical ventilation, *n* (%)	21 (9.5)	3 (1.6)	18 (51.4)	<0.001
CRRT, *n* (%)	10 (4.5)	1 (0.5)	9 (25.7)	<0.001
ECMO, *n* (%)	2 (0.9)	0 (0.0)	2 (5.7)	0.001
Clinical outcome				
Death, *n* (%)	21 (9.5)	2 (1.1)	19 (54.3)	<0.001

*AST, aspartate aminotransferase; AKI, acute kidney injury; ALF, acute liver failure; ARDS, acute respiratory distress syndrome; LMWH, low molecular weight heparin; IVGc, intravenous glucocorticoids; CRRT, continuous renal replacement therapy; ECMO, extracorporeal membrane oxygenation*.

Electrocardiograms were collected for 25 patients with cardiac injury. ST-T elevation was not found in any of the patients. Tachycardia was observed in all 25 patients. The next most common ECG abnormality was non-specific T-wave changes (32%). Atrial fibrillation was found in three patients. Limited bedside echocardiography was selectively performed in 24 patients. Among these patients, an ejection fraction <50% was observed in only one patient, and the most common finding was diastolic dysfunction (52%). Moderate tricuspid regurgitation was found in three patients.

With regard to treatment, the percentages of administering antiviral agents (20.05 vs. 7.0%, *P* < 0.001), glucocorticoids (51.4 vs. 23.5%, *P* = 0.001), inotropic agents (25.7 vs. 0.5%, *P* < 0.001), intravenous immunoglobulin (42.9 vs. 7.0%, *P* < 0.001), and low-molecular-weight heparin (31.4 vs. 2.1%, *P* < 0.001) were significantly higher in patients with elevated hs-TnI levels than in those with normal hs-TnI levels. Moreover, invasive and non-invasive mechanical ventilation (invasive: 51.4 vs. 1.6%, *P* < 0.001; non-invasive: 31.4 vs. 1.1%, *P* < 0.001) and continuous renal replacement therapy (25.7 vs. 0.5%, *P* < 0.001) were more frequently required in patients with cardiac injury than in those without cardiac injury. Notably, ECMO was used to support the most ill patients with cardiac injury among all of the included patients.

Among the 222 patients, 21 died during hospitalization, and 201 were discharged. A total of 19 (15/29 patients with cardiac injury on admission and 4/6 patients with new cardiac injury during hospitalization) of 35 (54.3%) patients died and 2 of 187 (1.1%) died in the cohorts with and without cardiac injury during hospitalization, respectively. Three patients suffered cardiac arrest before death. One patient died of ventricular fibrillation. All those four patients were in the group of cardiac injury.

**Figure 2:**
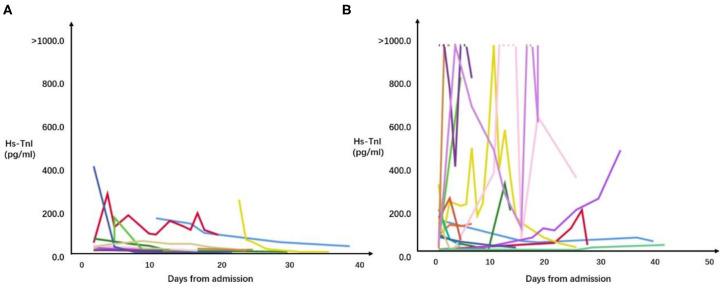
Correlations between results of all hs-TnI checked and time from admission in patients hospitalized with COVID-19 and cardiac injury. **(A)** Correlations between results of all hs-TnI checked and time from admission in survivors. **(B)** Correlations between results of all hs-TnI checked and time from admission in patients who died during hospitalization.

A total of 169 tests of hs-TnI were checked for patients with cardiac injury during hospitalization. The median of hs-TNI was 149.3 (IQR = 42.9–489.7) pg/mL in patients died during hospitalization and 29.0 (IQR = 15.0–90.6) pg/mL in the survivors. Correlations between results of hs-TnI and time from admission in patients with cardiac injury are shown in Figure 2. Among patients with cardiac injury, 17 patients had continuously increased levels of hs-TnI during hospitalization compared with the levels of hs-TnI on admission, and the mortality of these patients was significantly higher than those patients without continuously increased hs-TnI [13/17 (76.5%) vs. 6/18 (33.3%), *P* = 0.010].

### hs-TnI Levels on Admission and Predictors of Mortality in Patients With COVID-19 Infection

**Figure 3:**
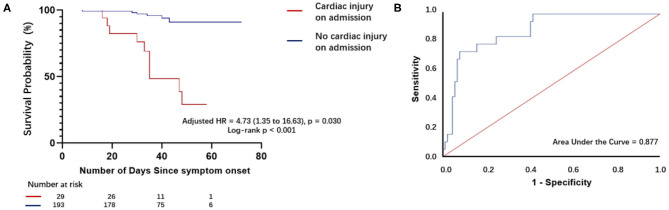
Survival curves of patients with COVID-19 with and without cardiac injury on admission and receiver operating characteristic curve analysis for plasma hs-TnI levels as a predictor of death. **(A)** Kaplan–Meier curves with cumulative hazards of death for comparison of patients with and those without cardiac injury. The mortality rate was higher in patients with cardiac injury on admission compared with those without cardiac injury on admission. **(B)** Receiver operating characteristic (ROC) curve and the area under the ROC curve for plasma hs-TnI levels on admission to predict death.

The mortality rate was higher in patients with elevated hs-TnI levels than in those with normal hs-TnI levels on admission (51.7 vs. 3.1%). Kaplan–Meier curves (Figure 3A) for mortality are shown in Figure 3 (log-rank test, *P* < 0.001). The multivariate Cox proportional hazards model showed that the risk of mortality was significantly higher in patients with elevated hs-TnI levels than in those with normal hs-TnI levels (adjusted HR = 4.73, 95% CI = 1.35–16.63, *P* = 0.015). Additionally, increased d-dimer levels (μg/mL) on admission were associated with a higher risk of mortality (adjusted HR = 1.10, 95% CI: 1.02–1.17, *P* = 0.011) (Table 3).

**Table 3:** Results of multivariable Cox regression analysis predicting death among 35 COVID-19 patients who had myocardial infarction at admission or during hospitalization vs. 187 COVID-19 patients who did not have myocardial infarction.

**Variable**	**Hazard ratio**	**95% CI**	***P-*value**
		**Lower**	**Upper**	
Evaluated hs-TnI	4.73	1.35	16.63	0.015
d-Dimer (μg/mL)	1.10	1.02	1.17	0.011

Receiver operating characteristic analysis (Figure 3B) for plasma hs-TnI levels showed good discriminatory power for mortality of patients with laboratory-confirmed SARS-CoV-2 infection. The area under the ROC curve was 0.880. The cutoff for plasma hs-TnI levels with the highest prognostic value was identified as 36.35 pg/mL. The sensitivity and specificity of the cutoff value were 73.7 and 92.1%, respectively.

## Discussion

The clinical course of the 222 patients with COVID-19 infection showed that cardiac injury was relatively common. A total of 13% of the patients had cardiac injury on admission, and 15.8% of patients had cardiac injury during the complete course of hospitalization. On admission, cardiac injury was more common in patients with hypoxemia. Troponin levels were correlated with the levels of inflammatory factors. These findings suggest that cardiac injury is most likely a secondary manifestation from respiratory distress and systemic infection. However, our findings could also be due to direct infection with the virus. During hospitalization, cardiac injury tended to be associated with multiorgan failure, more inotropic support and ventilator use, and consequent higher mortality. Along with elevated d-dimer levels, an increased troponin level up to 36 pg/mL on admission were predictive of death. Moreover, cardiac injury during hospitalization could, to some extent, become a confounding factor that contributes to mortality.

### Comparison With Previous Studies on COVID-19 From Wuhan

Our findings are consistent with the literature. Before the submission of our study, there have been five relatively large cohort studies that reported the incidence of cardiac injury and its association with mortality (3–7). We compared these studies with our study (Table 4). The mortality rate in our study was lower than that in most of the other studies (9.5% in our study vs. 4.3–28.3%). One of the reasons for this difference between studies is that our patients presented with less comorbidities than the other studies (less hypertension and less cardiac injury). Moreover, the timing of enrollment in the study played a major role in explaining the improved outcome in our study. Our study recruited patients who were admitted after February 10, 2020, when medical resources and staff were more readily available in Wuhan. However, the previous studies analyzed patients who were afflicted with COVID-19 in the early stage of the Wuhan epidemic.

**Table 4:** Previous studies which described reported incidence of cardiac injury and its association with mortality in COVID-19.

	**Study 1 (9)**	**Study 2 (6)**	**Study 3 (5)**	**Study 4 (4)**	**Study 5 (3)**	**Study 6 (24)**
First author	Shaobo Shi	Tao Guo	Dawei Wang	Fei Zhou	Tao Chen	Shaobo Shi
Publication date	3/25	3/27	2/7	3/9	3/26	5/6
Data source	Renmin Hospital of Wuhan University	Seventh Hospital of Wuhan City	Zhongnan Hospital of Wuhan University	Jinyintan Hospital and Wuhan Pulmonary Hospital	Tongji Hospital	Renmin Hospital of Wuhan University
Date of data collection	1/20–2/10	1/23–2/23	1/1–2/28	12/29–1/31	1/13–2/12	1/1–2/23
Sample size	416	187	138	191	274	671
Grouping	Cardiac injury (*n* = 82)/ramyano cardiac injury (*n* = 334)	Elevated TnT (*n* = 52)/normal TnT (*n* = 135)	ICU (*n* = 36)ramya/non-ICU (*n* = 102)	Death (*n* = 54)ramya/survivor (*n* = 137)	Deaths (*n* = 113)ramya/recovered (*n* = 161)	Death (*n* = 62)ramya/survivor (*n* = 609)
Study type	Cohort	Cohort	Descriptive	Case–control	Descriptive	Case–control
Overall age	64 (21–95)	58.50 (14.66)	56 (42–68)	56.0 (46.0–67.0)	62.0 (44.0–70.0)	63 (50–72)
Grouping age(year)	74 (34–95)/ramya60 (21–90)	71.40 (9.43)/ramya53.53 (13.22)	66 (57–78)/ramya51 (37–62)	69.0 (63.0–76.0)/ramya52.0 (45.0–58.0)	68.0 (62.0–77.0)/ramya51.0 (37.0–66.0)	74 (66–81)/ramya61 (49–70)
Overall hypertension (percentage)	30.5	32.6	31.2	30	34	29.7
Grouping hypertension (percentage)	59.8/23.4	63.5/20.7	58.3/21.6	48/23	48/24	59.7/26.6
Overall DM (percentage)	14.4	15.0	10.1	19	17	14.5
Grouping DM (percentage)	24.4/12.0	30.8/8.9	22.2/5.9	31/14	21/14	27.7/13.1
Overall CHD (percentage)	10.6	11.2	—	8	—	8.9
CHD (percentage)	29.3/6.0	32.7/3.0	—	24/1	—	33.9/6.4
Overall COPD (percentage)	2.9	2.1	2.9	3	—	3.4
COPD (percentage)	7.3/1.8	7.7/0	8.3/1.0	7/1	—	3.2/3.4
hs-TNI (pg/mL)	190 (80–1,120)/ <6 (<6–9)	—	11.0 (5.6–26.4)/5.1 (2.1–9.8)	22.2 (5.6–83.1)/3.0 (1.1–5.5)	40.8 (14.7–157.8)/3.3 (1.9–7.0)	0.235 (0.042–1.996)/0.006 (0.006–0.011)
Cardiac injury (percentage)	19.7	27.8	—	—	—	75.8/9.7
Overall mortality (percentage)	13.7	23	4.3	28.3	14.1	9.2
Grouping mortality (percentage)	51.2/4.5	59.6/8.9	—	—	—	—

*COVID-19, coronavirus disease 2019; DM, diabetes mellitus; CHD, chronic heart disease; COPD, chronic obstructive pulmonary disease; hs-Tni, high-sensitivity troponin I*.

### Possible Mechanism of Cardiac Injury

Cardiac injury is commonly found in patients with viral infection, and therefore, it is not unique for COVID-19 infection. Cardiac injury was found in 63.2% of patients who were infected with influenza A (H7N9) virus in China in the 2015–2017 outbreak, and these patients were associated with a high mortality rate (11). During the SARS and Middle East respiratory syndrome outbreaks, evidence of cardiac involvement was also reported (12, 13).

Cardiac injury in COVID-19 infection could be due to many reasons. Several case reports have shown acute myocardial infarction (8), acute myocarditis (14), acute myopericarditis (15), reverse takotsubo syndrome (16), and pulmonary embolism (17) as the culprits for cardiac injury. However, cardiac injury in our cohort appeared to be due to demand ischemia in response to overwhelming inflammation and/or hypoxia. Elevation of troponin levels is proportional to the levels of inflammatory factors, among which IL-6 is the most significant predictive factor for prognosis.

Cardiac injury in the setting of COVID-19 infection appears to follow a different pattern from what is known in typical acute viral myocarditis, which usually occurs after 1 week of viral infection. COVID-19–infected patients show evidence of cardiac injury only a couple of days after diagnosis of pneumonia. Additionally, abnormal electrical conduction or deadly arrhythmia is much more common in patients with acute viral myocarditis than in COVID-19–infected patients in whom tachycardia is most frequently observed. Although our study and previous studies showed that malignant arrhythmia, especially cardiac arrest, may occur at the end of the disease owing to severe hypoxia or electrolyte disorder. Moreover, in contrast to fulminant myocarditis, circulatory support was rarely required in our cohort. A pathological report showed pronounced pulmonary edema with hyaline membrane formation in the lungs in those who died of ARDS due to COVID-19. However, there were no obvious histological changes in cardiac tissue, as reported in a previous case report (18). Endomyocardial biopsy from one patient with COVID-19 who presented with cardiogenic shock showed only low-grade myocardial inflammation, which was inconsistent with fulminant myocarditis (19). Coronavirus particles were also found from the biopsy, which suggested either transient viremia or infected macrophage migration from the lungs.

COVID-19 infection is distinguished from bacterial sepsis by showing more cardiac involvement than other organs, such as renal or liver impairment. There are several explanations for such a discrepancy. A previous study showed that SARS-CoV-2 shared angiotensin-converting enzyme 2 (ACE2) as the host cellular receptor for virus spike (S) protein, and the expression and distribution of ACE2 were key determinants for entry of the virus (20). Patients who suffer from heart failure at baseline have increased ACE2 expression at mRNA and protein levels. If patients are infected by SARS-CoV-2, they might have a higher risk of adverse cardiac events (e.g., a heart attack and becoming critically ill). Paradoxically, older individuals, especially those with preexisting cardiovascular comorbidities, are more susceptible and succumb to the more severe form of COVID-19 infection, even though ACE2 expression is notably reduced with aging. This seemingly implausible observation could be argued by emphasizing the other perspective of ACE function, of which upregulation could lead to a positive immune response (21).

### Treatment Strategy of Cardiac Injury in Patients With COVID-19

**Figure 4:**
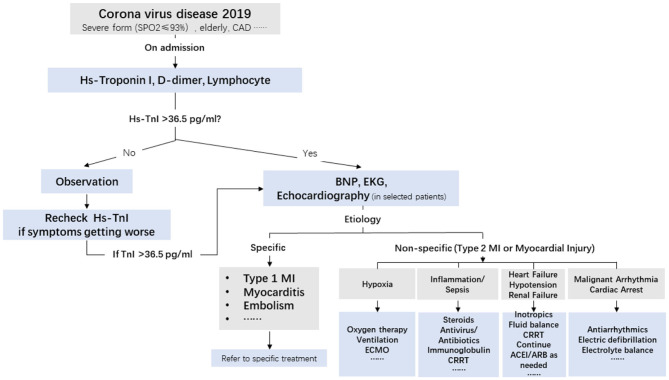
Triage and treatment of patients with COVID-19 based on hs-TnI levels at admission and the etiology of cardiac injury. CAD, coronary artery disease; BNP, B-type natriuretic peptide; ECG, electrocardiogram; MI, myocardial infarction; CRRT, continuous renal replacement therapy; ECMO, extracorporeal membrane oxygenation; hs-TnI, high-sensitivity troponin I.

Despite the fact that cardiac injury in COVID-19 infection is secondary to hypoxia and an inflammatory storm, cardiac involvement can portend a worse outcome and deteriorate the general well-being of infected patients. An example of this situation is that a rise in BNP levels indicates that normal heart function has become compromised. When severe pneumonia leads to septic shock found in COVID-19 infection, managing fluid balance becomes important. Similarly, cardiac injury and myocardial suppression might further predispose patients to volume and pressure overload, which could further deteriorate into severe pulmonary edema and cardiogenic shock. Therefore, early detection and intervention are effective in preventing adverse cardiac events (Figure 4).

Fortunately, myocardial suppression is reversible upon improvement of underlying respiratory distress. Although antiviral medication was anecdotally used in the patients in our study, some medications were discontinued in the middle of the course because of side effects. Steroids were used only when patients were febrile or their overall condition became worse. A low dosage of steroids for 1 week is suggested, while oral conversion and tapering are not necessary. Supportive treatment, such as immunoglobulin infusion, is frequently used to boost the immune system. Theoretically, IL-6 antibody could be used, but to date, there is no evidence that it is effective. To minimize inflammation, oxygenation is important. Discrete use of NIPV and early and prompt conversion to mechanical ventilation, while weighing the balance of applying ECMO to indicated patients, are paramount for decreasing the mortality rate. Our study showed that the longest ECMO support can last as long as 30 days, and patients still have a chance for extubation.

In our patients, angiotensin receptor blockers/ACE inhibitors were used in 3.6% of patients. Currently, there is no recommendation for stopping angiotensin receptor blockers/ACE inhibitors, especially for patients with heart failure. Continued administration of angiotensin receptor blockers/ACE inhibitors is recommended (22). Anticoagulation medication is frequently used in patients with cardiac injury, and antiplatelet medication is used only for patients with underlying coronary artery disease.

A comprehensive flowchart of triage and treatment of patients with COVID-19 patients based on troponin levels at admission and the etiology of cardiac injury is shown in the Central Illustration. A recent study showed that type 2 myocardial infarction and cardiac injury were associated with increased long-term mortality (23). Therefore, long-term follow-up of patients with COVID-19 and cardiac injury is required.

### Study Limitations

A limitation of this study is its retrospective design, especially because we studied patients in an urgently constructed hospital for the Wuhan COVID-19 outbreak. Specific limitations are as follows. First, data collection, especially ECG and echocardiographic data, was not complete. Therefore, the rate of myocardial infarction (mainly type 2) could not be obtained. Second, based on available evidence of our cohort and other cohorts, cardiac injury is mainly due to an oxygen supply demand imbalance and an inflammatory response. A coronary angiogram is rarely required in this situation. Therefore, there was no evidence of coronary status. Third, most of the data regarding cardiac injury were from Wuhan, China. To date, data from Italy, Spain, and the United States are sparse: In view of a more advanced age and a higher mortality rate in those epidemic areas, better knowledge of the incidence of cardiac injury and its contribution to morbidity and mortality in COVID-19 may lead to an improved prognosis.

## Conclusions

Cardiac injury is not uncommon and is relatively typical for COVID-19. Although cardiac injury is a manifestation secondary to systemic infection or hypoxia, it can complicate the disease course by compromising the patient's general condition and prolonging the course. We recommend checking troponin levels at admission and during hospitalization for better monitoring, managing, and predicating the prognosis of patients with COVID-19, especially in the most severe patients.

## Perspectives

Competency in Medical Knowledge: Cardiac injury in COVID-19 is a manifestation secondary to hypoxia and systemic infection and further complicates the disease course and increases the mortality rate.

Competency in Patient Care: Troponin levels should be checked at admission and during hospitalization for triage, better monitoring, and managing patients with COVID-19, especially in the most severe patients.

Translational Outlook: Cardiac injury is common in severe cases of COVID-19, and long-term follow-up for patients who survive from the severe form of COVID-19 might be required.

## Data Availability Statement

The raw data supporting the conclusions of this article will be made available by the authors, without undue reservation.

## Ethics Statement

The protocol of this study was approved by the ethics committee of First teaching hospital of JiLin University. Written informed consent was waived by the Ethics Commission for emerging infectious diseases. The data was retrospectively collected by two examiners from electronic medical record, and mutually checked for the accuracy. All the data was analyzed by investigators who were blind to this study.

## Author Contributions

WL and YW designed the research. YZ and QT analyzed and interpreted the data. ZX performed the statistical analysis and wrote the manuscript. LW and GL critically revised the manuscript for key intellectual content. WL was responsible for the integrity of the work as a whole. All authors approved the final version of the manuscript. All authors contributed to the article and approved the submitted version.

## Conflict of Interest

The authors declare that the research was conducted in the absence of any commercial or financial relationships that could be construed as a potential conflict of interest.

## Abbreviations

COVID-19: coronavirus disease 2019
C-reactive protein: CRP
hs-TnI: high-sensitivity troponin I
MI: myocardial infarction
ECMO: extracorporeal membrane oxygenation
ARDS: acute respiratory distress syndrome
SARS-CoV-2: severe acute respiratory syndrome coronavirus 2
BNP: B-type natriuretic peptide
IL-6: interleukin 6
ECG: electrocardiogram
CRRT: continuous renal replacement therapy.
